# Characterization and Automatic Discrimination between Predominant Hypoperfusion and Hyperperfusion Stages of NPDR

**DOI:** 10.3390/jpm14090977

**Published:** 2024-09-14

**Authors:** Luís Mendes, Luísa Ribeiro, Inês Marques, Conceição Lobo, José Cunha-Vaz

**Affiliations:** AIBILI—Association for Innovation and Biomedical Research on Light and Image, 3000-548 Coimbra, Portugal; mlribeiro@aibili.pt (L.R.); ipmarques@aibili.pt (I.M.); clobo@aibili.pt (C.L.); cunhavaz@aibili.pt (J.C.-V.)

**Keywords:** nonproliferative diabetic retinopathy, hypoperfusion, hyperperfusion, staging, hemodynamic

## Abstract

Background/Objectives: Diabetic retinopathy (DR) is a common diabetes complication that can lead to blindness through vision-threatening complications like clinically significant macular edema and proliferative retinopathy. Identifying eyes at risk of progression using non-invasive methods could help develop targeted therapies to halt diabetic retinal disease progression. Methods: A set of 82 imaging and systemic features was used to characterize the progression of nonproliferative diabetic retinopathy (NPDR). These features include baseline measurements (static features) and those capturing the temporal dynamic behavior of these static features within one year (dynamic features). Interpretable models were trained to distinguish between eyes with Early Treatment Diabetic Retinopathy Study (ETDRS) level 35 and eyes with ETDRS levels 43–47. The data used in this research were collected from 109 diabetic type 2 patients (67.26 ± 2.70 years; diabetes duration 19.6 ± 7.26 years) and acquired over 2 years. Results: The characterization of the data indicates that NPDR progresses from an initial stage of hypoperfusion to a hyperperfusion response. The performance of the classification model using static features achieved an area under the curve (AUC) of the receiver operating characteristics equal to 0.84 ± 0.07, while the model using both static and dynamic features achieved an AUC of 0.91 ± 0.05. Conclusion: NPDR progresses through an initial hypoperfusion stage followed by a hyperperfusion response. Characterizing and automatically identifying this disease progression stage is valuable and necessary. The results indicate that achieving this goal is feasible, paving the way for the improved evaluation of progression risk and the development of better-targeted therapies to prevent vision-threatening complications.

## 1. Introduction

Diabetic retinopathy (DR) is a frequent complication of diabetes and, through its vision-threatening complications, i.e., clinically significant macular edema (CSME) and proliferative diabetic retinopathy (PDR), may lead to blindness. It is estimated that by 2045 there will be 783 million people worldwide affected by diabetes [1]. Considering that a third of people with diabetes have signs of diabetic retinopathy, with 10% developing vision-threatening retinopathy, it is one of the leading causes of blindness in working-age people [1]. The presence of nonproliferative retinopathy (NPDR) is identified by microvascular changes that are typically asymptomatic. NPDR progresses silently, without vision loss from the mild to moderate and severe stages. However, the progression of nonproliferative retinopathy to vision-threatening stages, PDR, and CSME with vision loss vary from individual to individual. The cumulative occurrence of rates of progression from mild nonproliferative to vision-threatening complications has been determined to be in the order of 14–16% [2]. However, when there is moderate to severe retinopathy, then progression to complications is in the order of 58% [2]. In any case, predicting which people with NPDR are at high risk for progression to vision loss remains a challenge. It is, therefore, of major relevance to identify the presence of retinopathy in diabetic patients and, when present, to identify the eyes that have the greatest risk of progression and the greatest potential to benefit from treatment [3].

Although there are other disease severity scales [4], the gold standard method to perform the staging of DR, Early Treatment Diabetic Retinopathy Study (ETDRS) grading, is based on the identification of a series of lesions, mostly associated with microvascular disease [5]. The grading is done by manual inspection of 7 color fundus images (CFP) that cover approximately 90° of the retina (35° each). The ETDRS severity scale concerning the risk of developing eye complications is not linear. Eyes classified as moderate NPDR (ETDRS level 47) have an 8.6% 1-year risk of developing high-risk PDR, while the next level of severity (ETDRS level 53) has a 45% risk of developing high-risk PDR [6].

In recent years, several advances in clinical ophthalmological devices have been incorporated into clinical practice, leading to improvements in the characterization of the morphological and vascular changes associated with DR [7,8]. Metrics extracted from optical coherence tomography angiography (OCT) and optical coherence tomography angiography (OCTA) are currently used in clinical practice and have enhanced the characterization of eye condition [9].

Based on OCTA results for ETDRS levels 35 and 43–47, our group demonstrated significant non-perfusion in both the superficial capillary plexus (SCP) and deep capillary plexus (DCP). The evolution over two years was predominantly observed in the DCP for both skeletonized vessel density (VD) and perfusion density (PD). The microaneurysm turnover (MAT) also plays a crucial role in distinguishing between the two ETDRS groups [10].

In this study, we characterize ETDRS levels 35 and 43–47 using a set of 82 imaging and systemic features. These features include baseline measurements, referred to as static features, and those that capture the temporal dynamic behavior of these static features within one year, referred to as dynamic features. The inclusion of well-referenced non-imaging features linked to the risk of DR progression [11,12], such as metabolic features, allowed for a better characterization of the ETDRS levels. The use of both static and dynamic features enables us to characterize the ETDRS levels based on the observed predominance of hypoperfusion and hyperperfusion stages. To enhance this characterization, we also considered the progression of these eyes over a span of two years.

We also trained automatic interpretable models to distinguish between ETDRS 35 eyes, which exhibit predominant hypoperfusion, and ETDRS level 43–47 eyes, which exhibit predominant hyperperfusion. The performance of these models was evaluated using both static features alone and a combination of static and dynamic features. This approach allowed us to assess the impact of including dynamic behavior in the model inputs.

## 2. Materials and Methods

The data used in this research were collected from 109 diabetic type 2 patients (67.26 ± 2.70 years; diabetes duration 19.6 ± 7.26 years; 84 males, 25 females), acquired over 2 years under the CORDIS study (NCT03696810). For each patient, only one eye was included. The data include 73 eyes at the baseline, classified as ETDRS level 35, and 36 eyes as ETDRS level 43–47. The clinical study protocol underwent review and approval by the AIBILI Ethics Committee for Health, adhering to the principles outlined in the Declaration of Helsinki. Each participant received a detailed explanation of the informed consent form and provided their signature. Patients were excluded from participation if they had significant cataracts, confirmed glaucoma, recent eye surgery within the 6 months prior to the baseline visit, other retinal vascular diseases, prior laser treatments or intravitreal injections, or if their pupil dilation was less than 5 mm. Additional exclusion criteria included glycated hemoglobin A1C (HbA1c) levels exceeding 10% (85.8 mmol/mol) and other systemic conditions that could impact ocular health, with particular attention to uncontrolled systemic hypertension and a history of heart or kidney disease. Examinations occurred at baseline, 6 months, 12 months, and 24 months. All participants underwent ophthalmological assessments, including color fundus photography (CFP), OCT, and OCTA.

Seven-field CFP images were captured with a Topcon TRC-50DX mydriatic retinal camera (Topcon Medical Systems, Tokyo, Japan) at a 35° field of view. The resolution of these images was 3596 × 2448 pixels. Diabetic retinopathy severity grading followed the ETDRS protocol, focusing on the identification of specific lesions, including microaneurysms, hemorrhages, intraretinal microvascular abnormalities (IRMA), soft or hard exudates, venous beading, and neovascularization. The DR severity scores were determined at the Coimbra Ophthalmology Reading Centre (CORC) using the Diabetic Retinopathy Severity Scoring System.

OCTA examinations were performed using the Cirrus Zeiss 5000 AngioPlex (Zeiss, Dublin, CA, USA) with both the 3 × 3 mm and 6 × 6 mm angiography acquisition protocols during each visit. The 6 × 6 mm angiography protocol consists of 350 clusters of 2 B-scan repetitions with 350 A-scans, providing a wider field of view but lower digital sampling compared to the 3 × 3 mm angiography protocol, which includes 245 clusters of 4 B-scan repetitions with 245 A-scans.

The acquired 3 × 3 mm and 6 × 6 mm OCTA examinations were processed using the Carl Zeiss Meditec Density Exerciser (version: 10.0.12787; Carl Zeiss Meditec, Inc., Dublin, CA, USA) to compute the VD and PD metrics in the SCP and DCP. An OCTA quality check was performed by a masked grader to ensure that the signal strength was greater than or equal to 7 and that areas with motion artifacts, defocus, or blur were not present in more than 25% of the image area.

### 2.1. Feature Space

In this research work, a pool of 82 features (static and dynamic) was used to characterize ETDRS levels 35 and 43–47. In Table A1, Table A2, Table A3 and Table A4 of the annex presents a list of these features organized by static individual, static composed, dynamic individual, and dynamic composed features. Data preparation and cleaning were performed using the Julia language (version 1.10) and its associated ecosystem (DataFrames.jl, CSV.jl, GLM.jl).

#### 2.1.1. Static Features

Individual features

- OCT metrics

The central retinal thickness (RT_CSF), the ganglion cell layer (GCL) thickness measured in the inner ring (RT_InRing_GCL), and the average thickness of the GCL in the retina (RT_GCL) were measured using the Macular Cube 512 × 128 (512 A-scans with 128 B-scans each) acquisition protocol available on the Cirrus HD-OCT 5000 AngioPlex (Zeiss, Dublin, CA, USA).

- OCTA features

The VD and PD metrics in the SCP and DCP were computed using the Carl Zeiss Meditec Density Exerciser (version: 10.0.12787; Carl Zeiss Meditec, Inc., Dublin, CA, USA) for both the 3 × 3 mm and 6 × 6 mm OCTA acquisition protocols. The area and the number of intercapillary spaces (ICS) were calculated using the method previously described by our group [13]. The ICS metrics were computed based on a method that explores two established image processing methods, the bottom-hat transform and the closing morphological operator, to enhance the spaces of the binary version of the en face image generated by the Carl Zeiss Meditec Density Exerciser.

- Color Fundus Photography

Microaneurysm (MA) classification was automatically performed on 50° field 2 images using RetmarkerDR, a computer-aided diagnostic software developed by Retmarker SA (Meteda Group, Rome, Italy). The number of MAs counted automatically by RetmarkerDR at baseline was used in this research work.

- Systemic

Systemic parameters, including the duration of diabetes, body mass index, HbA1c level, total cholesterol, high-density lipoprotein (HDL), low-density lipoprotein (LDL), triglycerides, and blood pressure levels, were recorded at baseline.

Composed features

Composed features, computed using two individual features, were designed to encode the joint behavior of both. To encode into a single feature the different behaviors of the same feature measured at different spatial positions it was computed the ratio between the SCP and the DCP values (÷[Sp]) for the VD, PD, and ICS. To highlight the morphological changes associated with the same spatial position, the ratio between the values of VD and PD (÷[VD_PD]) for the different spatial areas was computed, also in addition to the ratio between the total area and the number of areas of ICS (÷[A_#]_ICS).

#### 2.1.2. Dynamic Features

The temporal dynamic behavior of the static OCTA features plus OCTA foveal avascular zone (FAZ) metrics (circularity, perimeter, and area) were also measured using OCTA and incorporated into the models by measuring the slope of the linear regression using three temporal points (baseline, 6 months, and 12 months). For ICS features, the slope was measured using only data from the baseline and 12 months. Data imputation using the Multivariate Imputation by Chained Equations (MICE) method [14] was employed to fill in missing values before measuring the slope values.

From CFP, the Retmarker DR was used to compute the MAT at 6 and 12 months. Additionally, apart from the automatic count of MAs, the Retmarker DR also calculates the rates of MA disappearance and formation across two visits. Microaneurysm turnover was determined as the sum of the MA formation and disappearance rates.

### 2.2. Statistic Data Analysis

Statistical analysis was performed using the Julia language (julialang.org; version 1.10) and associated packages (Statistics.jl, Distributions.jl, summaryTables.jl). The tables with the statistical analysis report the mean, standard deviation, median, maximum value, minimum value, and number of missing values. The missing values occurred when the value of a particular feature could not be considered for this study. For the image-based features, this could happen because the quality of the image was not sufficient for the feature to be computed. The values presented in the discussion are the median and the minimum and maximum values using the following format: median (minimum; maximum).

The *p*-values of the Mann–Whitney U test were utilized to determine whether the separation between groups was statistically significant. The Mann–Whitney U test is a non-parametric statistical test that allows testing if two samples originate from the same distribution. No assumption related to the distribution of the data is made. In this work, *p*-values < 0.05 were considered statistically significant. Additionally, the *p*-values were used to check if the separation of the values of the features measured between eyes that either improved or worsened over a 2-year follow-up period was statistically significant.

### 2.3. Classification Models and Performance Evaluation

Logistic regression (LR) models were trained to discriminate between ETDTS levels.

LR is a statistical method that can be used to perform binary classification. The method models the log probabilities of each class with a linear combination of numerical features, allowing for the prediction of which of two possible categories a data point belongs to. It estimates the probability of an outcome using the logistic function, which maps input features to a value between 0 and 1. By setting a threshold, the model classifies inputs into one of the two categories based on the estimated probability. The development and performance assessment were conducted using the Julia language (julialang.org; version 1.10) and its associated ecosystem (MLJ.jl, MLJLinear-Models.jl, Imbalance.jl, Impute.jl, RCall.jl).

Data imputation based on the k-nearest neighbors (KNN) algorithm [15] was also used during the training phase; i.e., data imputation using the KNN was used in the training dataset but not in the validation dataset. The Synthetic Minority Oversampling Technique (SMOTE) data augmentation method [16] was used to balance the training data.

The performance of the models was tested in three different scenarios: (1) when only one feature was used as an input to the models, (2) when static features were used as inputs, and (3) when static features were combined with dynamic features. For the last two scenarios, the forward feature selection (FFS) approach was employed to select up to five best features for model training. Performance metrics were measured after training the models using a repeated random sub-sampling validation approach (50 repetitions). In each task/dataset, the testing dataset comprised approximately 30% of the data, with a minimum of 10 samples from each class. The performance metrics included the area under the receiver operating characteristic curve (AUC), balance accuracy (bACC), sensitivity, and specificity.

## 3. Results

### 3.1. Characterization of ETDRS 35 and ETDRS 43–47

Concerning the 45 static features, only the number of MAs, with the values associated with ETDRS level 35 equal to 0 (0; 7) and those of ETDRS levels 43–47 equal to 3 (0; 13), and the ratio between the values of VD and PD measured at the inner ring in the deep capillary plexus, denoted as ÷[VD_PD]_InR_DCP, with the values of ETDRS level 35 equal to 51.7 (46.5; 54) and ETDRS levels 43–47 equal to 50.7 (48.6; 53.1), allow for a significant separation between ETDRS groups (Table 1).

For the 21 dynamic individual features (top of Table 2), the MAT measured at 6 months and 12 months, the values of the slopes associated with the VD and PD measured in the inner ring in both the SCP and DCP, and the FAZ circularity allow for a significant separation between groups.

**Table 1:** Descriptive statistics for the static features (individual and composed) of the ETDRS 35 and ETDRS 43–47 groups reveal statistically significant differences (with *p*-values < 0.05). Additionally, descriptive statistics are provided for two other features that approach the significance threshold of 0.05. These features are used to characterize the stages of NPDR and associated compensatory mechanisms. The n refers to the number of eyes.

	ETDRS			*p*-Value
	Overall (n = 109)	ETDRS35 (n = 73)	ETDRS43–47 (n = 36)	
**MA (#)**				<0.001
Mean (SD)	1.82 (2.53)	0.97 (1.52)	3.53 (3.24)	
Median [Min, Max]	1 [0, 13]	0 [0, 7]	3 [0, 13]	
**areaICS_DCP (pixeis)**				0.06
Mean (SD)	43872 (14936)	41647 (12880)	48131 (17670)	
Median [Min, Max]	43017 [14458, 115128]	43065 [14458, 85925]	42805 [18574, 115128]	
Missing	7 (6.42%)	6 (8.22%)	1 (2.78%)	
**÷[VD_PD]_InR_DCP (a.u)**				0.02
Mean (SD)	51.4 (1.28)	51.6 (1.27)	51 (1.23)	
Median [Min, Max]	51.5 [46.5, 54]	51.7 [46.5, 54]	50.7 [48.6, 53.1]	
Missing	7 (6.42%)	6 (8.22%)	1 (2.78%)	
**÷[A_#]_ICS_SCP (pixeis/#)**				0.069
Mean (SD)	250 (49.5)	244 (47.5)	261 (51.9)	
Median [Min, Max]	245 [168, 437]	243 [168, 437]	258 [169, 395]	
Missing	7 (6.42%)	6 (8.22%)	1 (2.78%)	

Notation: MA—microaneurysm; areaICS—area of intercapillary spaces (ICS); VD—skeletonized vessel density; PD—perfusion density; SCP—superficial capillary plexus; DCP—deep capillary plexus; InR—inner ring; ÷[A_#]—between the total area and the number of areas of ICS.

**Table 2:** Descriptive statistics for the dynamic features (individual and composed) of the ETDRS 35 and ETDRS 43–47 groups reveal statistically significant differences (with *p*-values < 0.05). The n refers to the number of eyes.

	ETDRS			*p*-Value
	Overall (n = 109)	ETDRS35 (n = 73)	ETDRS43–47 (n = 36)	
**MAT_6_Months** (no. per 6 months)				<0.001
Mean (SD)	5.21 (6.51)	3.24 (4.34)	9.21 (8.22)	
Median [Min, Max]	4 [0, 33.3]	2 [0, 21.8]	6.4 [0, 33.3]	
**MAT_12_Months** (no. per 12 months)				<0.001
Mean (SD)	3.05 (4.16)	1.5 (2.02)	6.2 (5.46)	
Median [Min, Max]	1 [0, 16.7]	1 [0, 8.3]	4.95 [0, 16.7]	
**//_VD_InR_DCP** (mm^−1^/6 months)				0.015
Mean (SD)	−0.391 (1.09)	−0.207 (0.992)	−0.764 (1.19)	
Median [Min, Max]	−0.373 [−2.86, 1.94]	−0.265 [−2.86, 1.94]	−1.0 [−2.66, 1.5]	
**//_VD_InR_SCP** (mm^−1^/6 months)				0.024
Mean (SD)	0.00964 (0.512)	0.0949 (0.508)	−0.163 (0.483)	
Median [Min, Max]	−0.032 [−1.2, 1.68]	0.0492 [−0.903, 1.68]	−0.182 [−1.2, 0.948]	
**//_FAZCircularity**				0.003
Mean (SD)	−0.00768 (0.0623)	0.0066 (0.0487)	−0.0366 (0.0763)	
Median [Min, Max]	−0.00244 [−0.268, 0.154]	0.0039 [−0.126, 0.154]	−0.0225 [−0.268, 0.108]	
**//_PD_InR_DCP** (a.u./6 months)				<0.001
Mean (SD)	−0.00805 (0.019)	−0.004 (0.0177)	−0.0162 (0.019)	
Median [Min, Max]	−0.00854 [−0.0479, 0.0367]	−0.0058 [−0.048, 0.037]	−0.0176 [−0.046, 0.031]	
**//_PD_InR_SCP** (a.u/6 months)				0.023
Mean (SD)	−0.00338 (0.0113)	−0.00164 (0.0107)	−0.00689 (0.0117)	
Median [Min, Max]	−0.00358 [−0.029, 0.037]	−0.0024 [−0.026, 0.036]	−0.00707 [−0.029, 0.025]	
**//_÷[SCP_DCP]_VD_InR_6 × 6** (a.u./6 months)				0.028
Mean (SD)	0.049 (0.164)	0.025 (0.163)	0.0977 (0.157)	
Median [Min, Max]	0.0312 [−0.512, 0.527]	0.0158 [−0.512, 0.524]	0.0752 [−0.15, 0.527]	
**//_÷[SCP_DCP]_PD_InR** (a.u./6 months)				0.037
Mean (SD)	0.0294 (0.0778)	0.0162 (0.0686)	0.0562 (0.089)	
Median [Min, Max]	0.0137 [−0.186, 0.251]	0.0123 [−0.186, 0.211]	0.0412 [−0.14, 0.251]	
**//_÷[SCP_DCP]_PD_InR_6 × 6** (a.u./6 months)				0.019
Mean (SD)	0.0455 (0.191)	0.0136 (0.183)	0.11 (0.193)	
Median [Min, Max]	0.038 [−0.616, 0.65]	−0.00553 [−0.616, 0.502]	0.0823 [−0.193, 0.65]	
**//_÷[SCP_DCP]_PD_OutR_6 × 6** (a.u./6 months)				0.051
Mean (SD)	0.0343 (0.199)	0.00587 (0.195)	0.0919 (0.198)	
Median [Min, Max]	−0.0015 [−0.53, 0.73]	−0.0147 [−0.53, 0.73]	0.0535 [−0.27, 0.53]	
**//_÷[VD_PD]_InR_DCP_6 × 6** (a.u./6 months)				0.033
Mean (SD)	0.103 (0.824)	−0.00455 (0.837)	0.32 (0.764)	
Median [Min, Max]	0.0868 [−1.91, 2.34]	0.0203 [−1.91, 2.34]	0.447 [−1.62, 1.82]	

Notation: //—slope measured between the baseline and the 12-month follow-up; MAT—microaneurysm turnover; VD—skeletonized vessel density; PD—perfusion density; SCP—superficial capillary plexus; DCP—deep capillary plexus; InR—inner ring; OutR—outer ring; 6 × 6—6 × 6 mm angiography acquisition protocol; ÷[SCP_DCP]—ratio between the values measured at the SCP and DCP; ÷[VD_PD]—ratio between the values of VD and PD.

Regarding the 16 dynamic composed metrics, (bottom of Table 2), only the slopes measured between the baseline and the 12-month mark for the ratio between the values measured at the SCP and DCP for PD measured in the inner ring using the 3×3 angiography acquisition protocol (//_÷[SCP_DCP]_PD_InR) and those measured in the inner ring and outer ring using the 6×6 angiography protocol (//_÷[SCP_DCP]_PD_InR_6 × 6, //_÷[SCP_DCP]_PD_OutR_6 × 6) allow for a significant separation between ETDRS groups. The slope of the ratio between the values measured at the SCP and DCP for VD in the inner ring (//_÷[SCP_DCP]_VD_InR_6 × 6), as well as the slope of the ratio measured between the baseline and the 12-month mark for the ratio between the VD and PD measured in the inner ring at the DCP (//_÷[VD_PD]_InR_DCP_6 × 6), also allow a significant separation between groups.

To enhance the characterization of the ETDRS 35 and ETDTS 43–37 levels, the progression of the ETDRS levels over a span of two years was also considered. Concerning the progression of eyes classified as ETDRS level 35 at baseline (Table 3), from the pull of 21 dynamic individual features, only the MAT measured at 6 months and 12 months and the values of the slopes associated with VD and PD (//_PD_InR_SCP, //_VD_InR_DCP_6 × 6, //_VD_OutR_DCP_6 × 6, //_PD_InR_DCP_6 × 6, //_PD_OutR_DCP_6 × 6) allow for a significant separation between eyes that improved and eyes that worsened within two years. Related with the progression of eyes classified as ETDRS levels 43–47 at baseline (Table 4), only the MAT and the slopes measured at 6 months and 12 months associated with the VD and PD measured in the inner ring at both the superficial capillary plexus and deep capillary plexus allow for a significant separation between groups.

### 3.2. Automated Discrimination between ETDRS 35 Eyes, Which Exhibit Predominant Hypoperfusion, and ETDRS 43–47 Eyes, Which Exhibit Hyperperfusion

Logistic regression models were trained with the objective of automatically discriminating between eyes classified as ETDSR level 35 and ETDSR levels 43–47, using a subset of a poll of static and dynamic features.

Table 5 presents the performance results of the trained models. At the top, we have the rank and performance of the 10 best models trained using only one feature. The first three places are models trained using features related to MAs (number of MAs and MA turnover measured at 6 and 12 months), and then comes the dynamic feature associated with FAZ circularity.

When the input of the model was MAs, we obtained an AUC equal to 0.8 ± 0.05, a bACC equal to 0.75 ± 0.07, a sensitivity equal to 0.7 ± 0.14, and a specificity equal to 0.79 ± 0.1. The middle of Table 5 presents the performance metrics of the models measured after training with one to five static features selected using the forward feature selection approach. The selected five features were MAs, PD_InR_SCP_6 × 6, triglycerides, ÷[VD_PD]_OutR_SCP_6 × 6, and RT_InR_GCL. With these five features, we obtained an AUC equal to 0.84 ± 0.07, a bACC equal to 0.82 ± 0.08, a sensitivity equal to 0.81 ± 0.13, and a specificity equal to 0.83 ± 0.12.

The bottom of Table 5 presents the performance metrics of the models measured after training with one to five static plus dynamic features selected using the forward feature selection approach. The selected five features were MAs, FAZ circularity, //_PD_InR_DCP_6 × 6, ÷[SCP_DCP]_PD_OutR_6 × 6, and ÷ [VD_PD]_InR_SCP_6 × 6. With these five features, we obtained an AUC equal to 0.91 ± 0.05, a bACC equal to 0.84 ± 0.08, a sensitivity equal to 0.84 ± 0.12, and a specificity equal to 0.83 ± 0.11.

## 4. Discussion

In this research work, a set of 82 features, which include both static features and features that capture their dynamic behavior within one year, was used to characterize changes in the compensatory hemodynamic mechanisms associated with ETDTS 35 (mild retinopathy) and ETDTS 43–47 (moderate severe retinopathy) and also to train models that allow for automated discrimination between these ETDRS severity grades. These 82 features were organized into four different groups: 29 static individual features, 16 static composed features, 21 dynamic individual features, and 16 dynamic composed features. To enhance the characterization, we also considered the progression of these eyes over a span of two years. The use of a large set of static and dynamic interpretable features has allowed us to characterize ETDRS stage 35 and ETDRS stages 43–47 based on the observed predominance of hypoperfusion and hyperperfusion stages. The existence of these stages is predicted by the unifying hemodynamic model for the pathogenesis of diabetic retinopathy proposed by Curtis et al. [17]. In the early stages, hypoperfusion may be a low-grade, chronic inflammation of the retinal vasculature, leading to capillary dropout and the development of progressive, irreversible ischemic hypoxia. According to Curtis’s model, as diabetes develops, increased hypoxia leads to hyperperfusion, which manifests as retinal vasodilation, increased retinal blood flow, and an abnormal number of microaneurysms [17].

The two static features that allow significative discrimination between the ETDRS groups were MAs and ÷[VD_PD]_InR_DCP (Table 1). For MAs, the median (min; max) value was 0 (0; 7) for the ETDRS 35 group and 3 (0; 13) for the ETDRS 43–47 group. The measured value for ÷[VD_PD]_InR_DCP was equal to 51.7 (46.5; 54) for the ETDRS 35 group and 50.7 (48.6; 53.1) for the ETDRS 43–47 group. These results support the hypothesis that hypoperfusion is associated with the ETDRS 35 level. The higher median value observed at the ETDRS 35 level suggests the presence of changes that are indicative of hypoperfusion, such as a reduction in vessel caliber and/or a decrease in the number of vessels. Furthermore, the median value associated with the area of ICS in the DCP (43,065 for ETDRS 35 and 42,805 for ETDRS 43–47) also supports this conclusion. These results are in line with the compensatory model proposed by Curtis et al. [17].

Considering dynamic features, hypoperfusion is characterized by a negative slope associated with VD_InR_DCP (//_VD_InR_DCP equal to −0.265 (−2.86; 1.94)). This corresponds to a decrease in the values of VD_InR_DCP over one year. This rate is also slower than in the ETDRS 43–47 group (//_VD_InR_DCP equal to −1.0 (−2.66; 1.5)). Also, for ETDRS level 35, there is a slight increase in VD_InR_SCP, a minor decrease in PD_InR_SCP, and a slight increase in the ratio of VD_PD in InR_SCP (÷[VD_PD]_InR_SCP). Further supporting the presence of predominant hypoperfusion in ETDRS level 35 is the increase in the value of the slope associated with ÷[VD_PD]_InR_SCP, with a magnitude ~85% lower (1 − (0.049/(0.265 + 0.049))) than the magnitude of the negative slope associated with ÷[VD_PD]_InR_DCP. These findings suggest that a compensatory mechanism is attempting to reverse the hypoperfusion in the SCP, but it is unsuccessful in the DCP. The success of this compensatory mechanism in the SCP may be attributed to the easier development of shutter vessels from the capillaries localized in the SCP due to the proximity of larger vessels. The behavior of FAZ circularity within one year (//_FAZCircularity) also follows these trends. The MA turnover values were also lower for the ETDRS 35 level.

A similar analysis can be performed now for the ETDRS level 43–47 group. For those levels, when compared with the hypoperfusion identified in the ETDRS 35 group, the values of ÷[VD_PD]_InR_DCP, VD_InR_DCP, and PD_InR_DCP suggest the existence of a condition associated with hyperperfusion determined by the presence of dilated shunt vessels.

The analyses of the slope within one year (Table 2) indicate that vessel metrics that allow for significative discrimination between groups have a negative median value in both the SCP and DCP, suggesting continuous reduction values. With the decreased number of open vessels, the MA turnover is increased for the ETDRS 43–47 grade (4.95 (0; 16.7)) when compared with ETDRS 35 (1.0 (0; 8.3)). These two findings, alongside the formation of IRMA, support the concept that at ETDRS levels 43–47, we have a hyperperfusion stage where the vascular net in the DCP is progressively closed and the SCP network is reorganized with the development of dilated vascular elements (re-organization of the blood distribution), later converting into IRMA.

The analysis of the changes in ETDRS levels over two years for each group suggests a shift in compensatory mechanisms between ETDRS 35 and ETDRS 43–47 that can be detected over a one-year interval. Among the ETDRS 35 group (Table 3), 13 eyes improved and 10 worsened within two years. The median value of the improved eyes showed a positive slope associated with PD in the inner ring of the SCP, while a negative slope was observed in the worsening group (//_PD_InR_SCP with a *p*-value = 0.03). The opposite behavior was measured for the DCP.

The analysis of the ETDRS 43–47 group (Table 4) indicates that, regardless of changes in ETDRS levels over two years, the vascular density in the inner ring decreased over one year.

The overall analysis of the behavior of VD, PD, MAs, and FAZ suggests that both hypoperfusion and hyperperfusion may be present in all NPDR stages. However, there is a predominance of hypoperfusion in the initial stages and a predominance of hyperperfusion in the later stages. Automatically discriminating eyes based on the predominant stage of disease progression will enhance the detection of eyes at risk of progression in clinical settings, potentially benefiting from timely treatment. Additionally, in the context of clinical trials and drug development, distinguishing between eyes in the hypoperfusion versus the hyperperfusion stages is also relevant.

Logistic regression models were trained using state-of-the-art techniques to automatically discriminate between eyes classified as ETDRS 35 and ETDRS 43–47. The models used metrics measured at a single time point (static metrics) or both static and dynamic features encoding behavior within one year. In the top three positions for better individual performance metrics were the metrics associated with microaneurysms (both static and dynamic). Although MAs appear in the early stages of diabetic retinopathy, their presence (number and turnover) substantially increases for ETDRS 43–47. Using only the number of MAs as the input to the models resulted in an AUC of 0.8 + 0.06. To enhance model performance, additional features were added using the FFS approach. Models were trained based on static metrics alone and static metrics combined with dynamic metrics to study the effect on model performance of the addition of dynamic features.

When five static metrics were used, the AUC increased from 0.8 + 0.06 to 0.84 + 0.07. In addition to the MAs, the other four selected metrics were PD in the inner ring of the SCP (PD_InR_SCP_6 × 6), triglycerides, the ratio between VD and PD measured in the outer ring of the SCP (÷[VD_PD]_InR_SCP_6 × 6), and retinal thickness in the inner ring of the ganglion cell layer (RT_InRing_GCL). The automatic selection of OCTA metrics measured at the superficial capillary plexus (SCP) supports the concept that important changes occur in the organization of this vascular layer between ETDRS level 35 and ETDRS levels 43–47.

When five static and dynamic metrics were considered, the model performance improved from an AUC of 0.8 + 0.05 to 0.91 + 0.05. In addition to MAs, the other four included metrics were the slope of FAZ circularity (//_FAZCircularity), the slope of PD measured in the inner ring at the DCP (//_PD_InR_DCP_6 × 6), the ratio between the values measured at the SCP and DCP for PD measured in the outer ring (÷[SCP_DCP]_PD_OutR_6 × 6), and the slope of the ratio of the values measured for VD and PD measured in the inner ring at the SCP (//_÷[VD_PD]_InR_SCP_6 × 6). These results suggest that the addition of dynamic features measured within one year as inputs to the models allows for an improvement in the performance of the models.

Interpretable models allow not only for discrimination between ETDRS level 35 and levels 43–47 but also provide insights related to morphological and vascular changes and the dynamics of the compensatory mechanisms of NDPD.

The major limitation of this analysis is the small number of eyes included. The smaller dataset not only impacts the characterization of the ETDRS levels but also affects the performance of the classification models and the identification of relevant features. However, the fact that they belong to patients with relatively well-controlled type 2 diabetes can be seen as an advantage, as it minimizes the influence of systemic factors. The exclusion criterium of HbA1c higher than 10% may, however, limit the generalization of the findings of this research. Data collected in clinical settings, using non-invasive examination, appear to offer the opportunity to identify the progression stage of eyes with NPDR.

## 5. Conclusions

Our data show that nonproliferative diabetic retinopathy progresses through an initial stage with a predominance of hypoperfusion, followed by a hyperperfusion response. The characterization and automatic identification of this disease’s stage of progression remains valuable and necessary. Our results indicate that achieving this goal is feasible. It opens the door for improved evaluation of the risk of progression and the development of better-targeted therapies to halt the progression of diabetic retinal disease, in order to prevent vision-threatening complications.

## Figures and Tables

**Table 3:** Descriptive statistics for the dynamic individual features (measured over a one-year period) of the eyes classified as ETDRS 35, which improve and worsen within two years, revealing statistically significant differences (with *p*-values < 0.05) between the two groups. Additionally, descriptive statistics are provided for other features that were used to improve the characterization of the stages of NPDR and associated compensatory mechanisms. The n refers to the number of eyes.

	ETDRS			*p*-Value
	Overall (n = 23)	35_Improve (n = 13)	35_Worse (n = 10)	
**MAT 6 months** (no. per 6 months)				<0.001
Mean (SD)	2.84 (3.52)	0.615 (1.24)	5.74 (3.43)	
Median [Min, Max]	2.1 [0, 9.8]	0 [0, 3.8]	6.5 [0, 9.8]	
**MAT 12 months** (no. per 12 months)				0.008
Mean (SD)	1.97 (2.72)	0.546 (0.782)	3.81 (3.25)	
Median [Min, Max]	1 [0, 8.3]	0 [0, 2]	3.05 [0, 8.3]	
**//_PD_InR_SCP** (u.a/6 months)				0.03
Mean (SD)	−0.000907 (0.0104)	0.00289 (0.00958)	−0.00585 (0.00979)	
Median [Min, Max]	−0.00258 [−0.0149, 0.0185]	0.0068 [−0.0107, 0.0185]	−0.0093 [−0.0149, 0.0167]	
**//_PD_InR_DCP** (u.a./6 months)				0.215
Mean (SD)	−0.00429 (0.019)	−0.000866 (0.018)	−0.00873 (0.0202)	
Median [Min, Max]	−0.00598 [−0.0413, 0.0297]	−0.00156 [−0.0413, 0.0297]	−0.0146 [−0.0331, 0.0294]	
**//_VD_InR_DCP_6 × 6** (mm^−1^/6 months)				0.011
Mean (SD)	0.0471 (1.18)	−0.534 (1.13)	0.803 (0.77)	
Median [Min, Max]	0.11 [−2.14, 2.19]	−0.942 [−2.14, 1.36]	0.812 [−0.247, 2.19]	
**//_VD_OutR_DCP_6 × 6** (mm^−1^/6 months)				0.011
Mean (SD)	−0.455 (1.49)	−1.14 (1.27)	0.442 (1.31)	
Median [Min, Max]	−0.281 [−2.72, 2.03]	−1.5 [−2.72, 1.32]	0.75 [−2.12, 2.03]	
**//_PD_InR_DCP_6 × 6** (u.a./6 months)				0.002
Mean (SD)	−0.00471 (0.0305)	−0.0229 (0.0259)	0.019 (0.0168)	
Median [Min, Max]	−0.00348 [−0.0517, 0.046]	−0.0308 [−0.0517, 0.0326]	0.0201 [−0.00551, 0.046]	
**//_PD_OutR_DCP_6 × 6** (u.a./6 months)				0.009
Mean (SD)	−0.00259 (0.034)	−0.0194 (0.0319)	0.0192 (0.0233)	
Median [Min, Max]	−0.00491 [−0.057, 0.0435]	−0.0142 [−0.057, 0.035]	0.0231 [−0.0265, 0.0435]	

Notation: //—slope measured between the baseline and the 12-month follow-up; MAT—microaneurysm turnover; VD—skeletonized vessel density; PD—perfusion density; SCP—superficial capillary plexus; DCP—deep capillary plexus; InR—inner ring; OutR—outer ring; 6 × 6—6 × 6 mm angiography acquisition protocol.

**Table 4:** Descriptive statistics for the dynamic individual features (measured over one year) of eyes classified as ETDRS 43–47, which improve and worsen within two years, revealing statistically significant differences (with *p*-values < 0.05) between the two groups. Additionally, descriptive statistics are provided for other features that were used to improve the characterization of the stages of NPDR and associated compensatory mechanisms. The n refers to the number of eyes.

	ETDRS			*p*-Value
	Overall (n = 12)	43–47_Improve (n = 10)	43–47_Worse (n = 2)	
**MAT 6 months** (no. per 6 months)				0.03
Mean (SD)	7.43 (9.65)	3.75 (3.36)	25.8 (10.6)	
Median [Min, Max]	4.2 [0, 33.3]	4.1 [0, 10.4]	25.8 [18.3, 33.3]	
**MAT 12 months** (no. per 12 months)				0.028
Mean (SD)	3.88 (5.16)	2.08 (2.97)	12.9 (4.24)	
Median [Min, Max]	1.5 [0, 15.9]	1 [0, 9.4]	12.9 [9.9, 15.9]	
**//_VD_InR_DCP** (mm^−1^/6 months)				0.83
Mean (SD)	−0.943 (1.04)	−0.93 (0.978)	−1.01 (1.82)	
Median [Min, Max]	−1.01 [−2.39, 0.287]	−1.01 [−2.39, 0.287]	−1.01 [−2.29, 0.28]	
**//_VD_InR_SCP** (mm^−1^/6 months)				0.83
Mean (SD)	−0.216 (0.428)	−0.219 (0.47)	−0.202 (0.152)	
Median [Min, Max]	−0.178 [−0.899, 0.399]	−0.178 [−0.899, 0.399]	−0.202 [−0.31, −0.0949]	
**//_PD_InR_DCP** (u.a./6 months)				0.133
Mean (SD)	−0.0208 (0.0184)	−0.0175 (0.0182)	−0.0376 (0.00849)	
Median [Min, Max]	−0.0228 [−0.0464, 0.00452]	−0.0189 [−0.0464, 0.00452]	−0.0376 [−0.0436, −0.0316]	
**//_PD_InR_SCP** (u.a./6 months)				0.197
Mean (SD)	−0.0115 (0.011)	−0.00975 (0.0106)	−0.0203 (0.0114)	
Median [Min, Max]	−0.0104 [−0.0286, 0.00659]	−0.00878 [−0.0286, 0.00659]	−0.0203 [−0.0283, −0.0122]	

Notation: //—slope measured between the baseline and the 12-month follow-up; MAT—microaneurysm turnover; VD—skeletonized vessel density; PD—perfusion density; SCP—superficial capillary plexus; DCP—deep capillary plexus; InR—inner ring.

**Table 5:** Performance results of the models trained to discriminate between 35 and 43–47. At the top, we have the rank and performance of the models associated with the top 10 features, measured after training using only one feature. In the middle and bottom, we see performance metrics of the models measured after training with one to five features selected using the forward feature selection approach. The middle section corresponds to when only static metrics were used, while the bottom section includes both static and dynamic metrics. For all the evaluations, a repeated random sub-sampling validation approach was employed (50 repetitions). Each task/dataset had a testing dataset with 30% of the data, ensuring a minimum of 10 eyes of each class in the testing set.

**Individual Features Performance**						
**Rank**	**Feature**		**AUC**	**bACC**	**Sensitivity**	**Specificity**
1	MAs		0.8 ± 0.06	0.75 ± 0.07	0.7 ± 0.14	0.79 ± 0.1
2	MAT_6_months		0.8 ± 0.06	0.7 ± 0.07	0.68 ± 0.11	0.72 ± 0.1
3	MAT_12_months		0.79 ± 0.07	0.75 ± 0.06	0.66 ± 0.12	0.85 ± 0.09
4	//_FAZCircularity		0.79 ± 0.07	0.71 ± 0.07	0.75 ± 0.11	0.67 ± 0.14
5	//_PD_InR_DCP		0.72 ± 0.07	0.67 ± 0.07	0.67 ± 0.12	0.67 ± 0.13
6	//_÷[SCP_DCP]_PD_InR		0.71 ± 0.08	0.66 ± 0.08	0.64 ± 0.12	0.67 ± 0.12
7	//_FAZPerimeter		0.69 ± 0.12	0.62 ± 0.1	0.69 ± 0.16	0.55 ± 0.19
8	//_ VD_InR_DCP		0.68 ± 0.08	0.64 ± 0.06	0.66 ± 0.11	0.61 ± 0.12
9	÷[VD_PD]_InR_DCP		0.65 ± 0.1	0.6 ± 0.1	0.56 ± 0.15	0.64 ± 0.15
10	//_÷[SCP_DCP]_VD_InR		0.64 ± 0.11	0.61 ± 0.09	0.62 ± 0.14	0.61 ± 0.15
**FFS Performance - Static features**						
**#features**	**AUC**	**bACC**	**Sensitivity**	**Specificity**	**Features**	
1	0.8 ± 0.06	0.75 ± 0.07	0.7 ± 0.14	0.79 ± 0.1	MAs	
2	0.84 ± 0.07	0.79 ± 0.07	0.76 ± 0.14	0.82 ± 0.1	+ PD_InR_SCP_6 × 6	
3	0.84 ± 0.07	0.81 ± 0.08	0.79 ± 0.13	0.83 ± 0.12	+ triglycerides	
4	0.84 ± 0.07	0.81 ± 0.08	0.8 ± 0.14	0.83 ± 0.11	+ ÷[VD_PD]_OutR_SCP_6 × 6	
5	0.84 ± 0.07	0.82 ± 0.08	0.81 ± 0.13	0.83 ± 0.12	+ RT_Inr_GCL	
**FFS Performance - Static + Dynamic features**						
**#features**	**AUC**	**bACC**	**Sensitivity**	**Specificity**	**Features**	
1	0.8 ± 0.06	0.75 ± 0.07	0.7 ± 0.14	0.79 ± 0.1	MAs	
2	0.87 ± 0.05	0.78 ± 0.06	0.76 ± 0.12	0.79 ± 0.1	+ //_FAZCircularity	
3	0.89 ± 0.06	0.83 ± 0.06	0.83 ± 0.09	0.82 ± 0.11	+ //_ PD_InR_DCP_6 × 6	
4	0.91 ± 0.05	0.81 ± 0.09	0.81 ± 0.14	0.8 ± 0.12	+ ÷[SCP_DCP]_PD_OurR_6 × 6	
5	0.91 ± 0.05	0.84 ± 0.08	0.84 ± 0.12	0.83 ± 0.11	+ //_÷[VD_PD]_InR_SCP_6 × 6	

Notation: //—slope measured between the baseline and the 12-month follow-up; MA—microaneurysm; MAT—microaneurysm turnover; VD—skeletonized vessel density; PD—perfusion density; SCP—superficial capillary plexus; DCP—deep capillary plexus; InR—inner ring; OutR—outer ring; 6 × 6—6 × 6 mm angiography acquisition protocol; ÷[SCP_DCP]—ratio between the values measured at the SCP and DCP; ÷[VD_PD]—ratio between the measured values of VD and PD; FAZCircularity—circularity of the foveal avascular zone; FAZPerimeter—perimeter of the foveal avascular zone.

## Data Availability

Data will be available upon on reasonable request to the correspondent author.

## Appendix A

**Table A1:** Static individual features.

Feature	Description
**CFP**	
MA	Number of micro-aneurisms at baseline
**OCT**	
RT_CSF	Retinal thickness in the central subfield
RT_GCL	Mean of the GCL thickness in the retina
RT_InRing_GCL	GCL thickness measured in the inner ring
**OCTA**	
areaICS_SCP	Number of pixels detected as capillary spaces in the SCP
areaICS_DCP	Number of pixels detected as capillary spaces in the DCP
nICS_SCP	Number of individual regions detected as capillary spaces in the SCP
nICS_DCP	Number of individual regions detected as capillary spaces in the DCP
VD_InR_SCP	Vessel density measured in the inner ring at the SCP (acquisition protocol angio 3 × 3 mm)
VD_InR_SCP_6 × 6	Vessel density measured in the inner ring at the SCP (acquisition protocol angio 6 × 6 mm)
VD_InR_DCP	Vessel density measured in the inner ring at the DCP (acquisition protocol angio 3 × 3 mm)
VD_InR_DCP_6 × 6	Vessel density measured in the inner ring at the DCP (acquisition protocol angio 6 × 6 mm)
PD_InR_SCP	Perfusion density measured in the inner ring at the SCP (acquisition protocol angio 3 × 3 mm)
PD_InR_SCP_6 × 6	Perfusion density measured in the inner ring at the SCP (acquisition protocol angio 6 × 6 mm)
PD_InR_DCP	Perfusion density measured in the inner ring at the DCP (acquisition protocol angio 3 × 3 mm)
PD_InR_DCP_6 × 6	Perfusion density measured in the inner ring at the DCP (acquisition protocol angio 6 × 6 mm)
VD_OutR_SCP_6 × 6	Vessel density measured in the inner ring at the SCP (acquisition protocol angio 6 × 6 mm)
VD_OutR_DCP_6 × 6	Vessel density measured in the inner ring at the DCP (acquisition protocol angio 6 × 6 mm)
PD_OutR_SCP_6 × 6	Perfusion density measured in the inner ring at the SCP (acquisition protocol angio 6 × 6 mm)
PD_OutR_DCP_6 × 6	Perfusion density measured in the inner ring at the DCP (acquisition protocol angio 6 × 6 mm)
**Systemic**	
bodyMassInd	Body mass measured at baseline
diabDuration	Years of diabetes duration at baseline
systBloodP	Systolic blood pressure measured at baseline
diastBloodP	Diastolic blood pressure measured at baseline
HbA1C	Glycated hemoglobin measured at baseline
totalCholest	Total cholesterol at baseline
ldlCholest	Total cholesterol at baseline
hdlCholest	Low-density lipoprotein cholesterol at baseline
Triglycerides	Triglycerides at baseline

**Table A2:** Static composed features.

OCTA Feature	Description
÷[SCP_DCP]_VD_InR	Ratio between the values measured at the SCP and DCP for the VD in the inner ring (acquisition protocol 3 × 3 mm)
÷[SCP_DCP]_VD_InR_6 × 6	Ratio between the values measured at the SCP and DCP for the VD in the inner ring (acquisition protocol 6 × 6 mm)
÷[SCP_DCP]_PD_InR	Ratio between the values measured at the SCP and DCP for the PD in the inner ring (acquisition protocol 3 × 3 mm)
÷[SCP_DCP]_PD_InR_6 × 6	Ratio between the values measured at the SCP and DCP for the PD in the inner ring (acquisition protocol 6 × 6 mm)
÷[SCP_DCP]_VD_OutR_6 × 6	Ratio between the values measured at the SCP and DCP for the VD in the OutRing (acquisition protocol 6 × 6 mm)
÷[SCP_DCP]_PD_OutR_6 × 6	Ratio between the values measured at the SCP and DCP for the PD in the OutRing (acquisition protocol 6 × 6 mm)
÷[SCP_DCP]_areaICS	Ratio between the values measured at the SCP and DCP for the areaICS (acquisition protocol 3 × 3 mm)
÷[SCP_DCP]_nICS	Ratio between the values measured at the SCP and DCP for the nICS (acquisition protocol 3 × 3 mm)
÷[VD_PD]_InR_SCP	Ratio between the values of VD and PD measured at inner ring in SCP (acquisition protocol angio 3 × 3 mm)
÷[VD_PD]_InR_DCP	Ratio between the values of VD and PD measured at inner ring in DCP (acquisition protocol angio 3 × 3 mm)
÷[VD_PD]_InR_SCP_6 × 6	Ratio between the values of VD and PD measured at inner ring in SCP (acquisition protocol angio 6 × 6 mm)
÷[VD_PD]_InR_DCP_6 × 6	Ratio between the values of VD and PD measured at inner ring in DCP (acquisition protocol angio 6 × 6 mm)
÷[VD_PD]_OutR_SCP_6 × 6	Ratio between the values of VD and PD measured at Outer ring in SCP (acquisition protocol angio 3 × 3 mm)
÷[VD_PD]_OutR_DCP_6 × 6	Ratio between the values of VD and PD measured at Outer ring in DCP (acquisition protocol angio 3 × 3 mm)
÷[A_#]_ICS_SCP	Ratio between the values of area and number of regions of ICS at SCP (acquisition protocol angio 3 × 3 mm)
÷[A_#]_ICS_DCP	Ratio between the values of area and number of regions of ICS at DCP (acquisition protocol angio 3 × 3 mm)

**Table A3:** Dynamic individual features.

Features	Description
**CFP**	
MAT_6_months	Micro-aneurisms turnover measured between the baseline and 6 months
MAT_12_months	Micro-aneurisms turnover measured between the baseline and 12 months
**OCTA**	
//_VD_InR_SCP	Slope measured between the baseline and the 12-month follow-up for the VD_InR_SCP (acquisition protocol angio 3 × 3 mm)
//_VD_InR_DCP	Slope measured between the baseline and the 12-month follow-up for the VD_InR_DCP (acquisition protocol angio 3 × 3 mm)
//_PD_InR_SCP	Slope measured between the baseline and the 12-month follow-up for the PD_InR_SCP (acquisition protocol angio 3 × 3 mm)
//_PD_InR_DCP	Slope measured between the baseline and the 12-month follow-up for the PD_InR_DCP (acquisition protocol angio 3 × 3 mm)
//_VD_InR_SCP_6 × 6	Slope measured between the baseline and the 12-month follow-up for the VD_InR_SCP (acquisition protocol angio 6 × 6 mm)
//_VD_InR_DCP_6 × 6	Slope measured between the baseline and the 12-month follow-up for the VD_InR_DCP (acquisition protocol angio 6 × 6 mm)
//_PD_InR_SCP_6 × 6	Slope measured between the baseline and the 12-month follow-up for the PD_InR_SCP (acquisition protocol angio 6 × 6 mm)
//_PD_InR_DCP_6 × 6	Slope measured between the baseline and the 12-month follow-up for the PD_InR_DCP (acquisition protocol angio 6 × 6 mm)
//_VD_OutR_SCP_6 × 6	Slope measured between the baseline and the 12-month follow-up for the VD_OutR_SCP (acquisition protocol angio 6 × 6 mm)
//_VD_ OutR _DCP_6 × 6	Slope measured between the baseline and the 12-month follow-up for the VD_OutR_DCP (acquisition protocol angio 6 × 6 mm)
//_PD_ OutR _SCP_6 × 6	Slope measured between the baseline and the 12-month follow-up for the PD_OutR_SCP (acquisition protocol angio 6 × 6 mm)
//_PD_ OutR _DCP_6 × 6	Slope measured between the baseline and the 12-month follow-up for the PD_OutR_DCP (acquisition protocol angio 6 × 6 mm)
//_FAZArea	Slope measured between the baseline and the 12-month follow-up for the FAZArea (acquisition protocol angio 3 × 3 mm)
//_FAZPerimeter	Slope measured between the baseline and the 12-month follow-up for the FAZPerimeter (acquisition protocol angio 3 × 3 mm)
//_FAZCircularity	Slope measured between the baseline and the 12-month follow-up for the FAZCircularity (acquisition protocol angio 3 × 3 mm)
//_areaICS_SCP	Slope measured between the baseline and the 12-month follow-up for the areaICS_SCP (acquisition protocol angio 3 × 3 mm)
//_areaICS_DCP	Slope measured between the baseline and the 12-month follow-up for the areaICS_DCP (acquisition protocol angio 3 × 3 mm)
//_nICS_SCP	Slope measured between the baseline and the 12-month follow-up for the nICS_SCP (acquisition protocol angio 3 × 3 mm)
//_nICS_DCP	Slope measured between the baseline and the 12-month follow-up for the nICS_DCP (acquisition protocol angio 3 × 3 mm)

**Table A4:** Dynamic composed features.

OCTA Feature	Description
//_÷[SCP_DCP]_VD_InR	Slope measured between the baseline and the 12-month follow-up for the ÷[SCP_DCP]_VD_InR (acquisition protocol angio 3 × 3 mm)
//_÷[SCP_DCP]_VD_InR_6 × 6	Slope measured between the baseline and the 12-month follow-up for the ÷[SCP_DCP]_VD_InR (acquisition protocol angio 6 × 6 mm)
//_÷[SCP_DCP]_PD_InR	Slope measured between the baseline and the 12-month follow-up for the ÷[SCP_DCP]_VD_InR (acquisition protocol angio 3 × 3 mm)
//_÷[SCP_DCP]_PD_InR_6 × 6	Slope measured between the baseline and the 12-month follow-up for the ÷[SCP_DCP]_PD_InR_6 × 6 (acquisition protocol angio 6 × 6 mm)
//_÷[SCP_DCP]_VD_OutR_6 × 6	Slope measured between the baseline and the 12-month follow-up for the ÷[SCP_DCP]_VD_OutR_6 × 6 (acquisition protocol angio 6 × 6 mm)
//_÷[SCP_DCP]_PD_OutR_6 × 6	Slope measured between the baseline and the 12-month follow-up for the ÷[SCP_DCP]_PD_OutR_6 × 6 (acquisition protocol angio 6 × 6 mm)
//_÷[SCP_DCP]_areaICS	Slope measured between the baseline and the 12-month follow-up for the ÷[SCP_DCP]_areaICS (acquisition protocol angio 3 × 3 mm)
//_÷[SCP_DCP]_nICS	Slope measured between the baseline and the 12-month follow-up for the ÷[SCP_DCP]_nICS (acquisition protocol angio 3 × 3 mm)
//_÷[VD_PD]_InR_SCP	Slope measured between the baseline and the 12-month follow-up for the ÷[VD_PD]_InR_SCP (acquisition protocol angio 3 × 3 mm)
//_÷[VD_PD]_InR_DCP	Slope measured between the baseline and the 12-month follow-up for the ÷[VD_PD]_InR_DCP (acquisition protocol angio 3 × 3 mm)
//_÷[VD_PD]_InR_SCP_6 × 6	Slope measured between the baseline and the 12-month follow-up for the ÷[VD_PD]_InR_SCP_6 × 6 (acquisition protocol angio 6 × 6 mm)
//_÷[VD_PD]_InR_DCP_6 × 6	Slope measured between the baseline and the 12-month follow-up for the ÷[VD_PD]_InR_DCP_6 × 6 (acquisition protocol angio 6 × 6 mm)
//_÷[VD_PD]_OutR_SCP_6 × 6	Slope measured between the baseline and the 12-month follow-up for the ÷[VD_PD]_OutR_SCP_6 × 6(acquisition protocol angio 6 × 6 mm)
//_÷[VD_PD]_OutR_DCP_6 × 6	Slope measured between the baseline and the 12-month follow-up for the ÷[VD_PD]_OutR_DCP_6 × 6 (acquisition protocol angio 6 × 6 mm)
//_÷[A_#]_ICS_SCP	Slope measured between the baseline and the 12-month follow-up for the ÷[A_#]_ICS_SCP (acquisition protocol angio 3 × 3 mm)
//_÷[A_#]_ICS_DCP	Slope measured between the baseline and the 12-month follow-up for the ÷[A_#]_ICS_DCP (acquisition protocol angio 3 × 3 mm)
